# Previously Unidentified Single Nucleotide Polymorphisms in HIV/AIDS Cases Associate with Clinical Parameters and Disease Progression

**DOI:** 10.1155/2016/2742648

**Published:** 2016-12-05

**Authors:** Vladimir V. Anokhin, Liliia B. Bakhteeva, Gulshat R. Khasanova, Svetlana F. Khaiboullina, Ekaterina V. Martynova, Richard L. Tillett, Karen A. Schlauch, Vincent C. Lombardi, Albert A. Rizvanov

**Affiliations:** ^1^Kazan State Medical University, 49 Butlerova St., Kazan 420012, Russia; ^2^Republican Clinical Hospital for Infectious Disease named after Agafonov, 83 Prospect Pobedy St, Kazan 420140, Russia; ^3^Republican Center for the Prevention and Fighting of AIDS and Infectious Diseases, Ministry of Health of the Republic of Tatarstan, 2a Vishevskogo St., Kazan 420097, Russia; ^4^Kazan Federal University, 18 Kremlyovskaya St., Kazan 420008, Russia; ^5^Department of Biochemistry, University of Nevada, Reno, 1664 N. Virginia Street MS 0330, Reno, NV 89557, USA; ^6^Nevada Center for Biomedical Research, University of Nevada, Reno, 1664 N. Virginia Street MS 0552, Reno, NV 89557, USA

## Abstract

The genetic background of an individual plays an important role in the progression of HIV infection to AIDS. Identifying previously unknown or uncharacterized single nucleotide polymorphisms (SNPs) that associate with disease progression may reveal important therapeutic targets and provide a greater understanding of disease pathogenesis. In the present study, we employed ultra-high multiplex PCR on an Ion Torrent next-generation sequencing platform to sequence 23 innate immune genes from 94 individuals with HIV/AIDS. This data was used to identify potential associations of SNPs with clinical parameters and disease progression. SNPs that associated with an increased viral load were identified in the genes for the interleukin 15 receptor (*IL15RA*), toll-like receptor 7 (*TLR7*), tripartite motif-containing protein 5 (*TRIM5*), and two killer-cell immunoglobulin-like receptors (*KIR2DL1* and* KIR2DL3*). Additionally, SNPs that associated with progression from HIV infection to AIDS were identified in two 2′-5′-oligoadenylate synthetase genes (*OAS2* and* OAS3*). In contrast, other SNPs identified in* OAS2* and* OAS3* genes, as well as in the* TRIM5* and* KIR2DS4* genes, were associated with a slower progression of disease. Taken together, our data demonstrates the utility of ultra-high multiplex PCR in identifying polymorphisms of potential clinical significance and further,identifies SNPs that may play a role in HIV pathogenesis.

## 1. Introduction

Acquired immunodeficiency syndrome (AIDS) is a devastating disease caused by the human immunodeficiency virus (HIV). AIDS is defined as having a CD4^+^ T cell count below 200 cells per *μ*L or displaying specific clinical presentations in association with an HIV infection [1]. On average, most individuals infected with HIV will progress to AIDS within 10 years if antiretroviral therapy is not administered.

It is evident that the clinical manifestations of HIV infection vary in different individuals. Previous studies have reported that these differences are not dependent on gender, mode of infection, or age [2]. Therefore, genetic variations in genes that control immune responses have been suggested to play a role in the progression of HIV or lack thereof [3, 4]. Indeed, innate immunity plays an important role in controlling HIV replication [5]. Upregulation of type I interferon (IFN) is essential to restrain HIV replication, especially at the early stages of infection [6]. The activation of IFN*α* expression occurs primarily through the engagement of toll-like receptors (TLR) 7 and 9 by single stranded RNA or CpG DNA, respectively (reviewed by [7]). It has been demonstrated that single nucleotide polymorphisms (SNPs) in TLRs are associated with an increased susceptibility to infection [8–11] and are involved in different progressions of HIV/AIDS, although a consensus has not been reached. For example, Papadopoulos et al. demonstrated an association between a* TLR4* polymorphism and increased risk of opportunistic infection in subjects with advanced HIV-1 infection [8]. However, a subsequent study by Soriano-Sarabia et al. could not confirm these results, although these investigators did identify a polymorphism in* TLR9* that was associated with a lower CD4 count and a higher viral load in HIV-positive cases [12].

Numerous reports describe impaired immunomodulatory and cytotoxic activity of natural killer (NK) cells in HIV-1-infected individuals [5, 13–15]. Additionally, HIV infections are often characterized by increased expression of inhibitory receptors and downregulation of activating receptors [16, 17]. Killer-cell immunoglobulin-like receptors (KIRs) regulate NK activation status. Interestingly, NK cells expressing the activating KIR3DS1 inhibit HIV-1 replication in target cells [18]. Therefore, it could be suggested that differential expression of various KIRs may impact progression and outcome of HIV infection. Although the presence of some SNPs can alter the expression and function of KIRs, our knowledge regarding polymorphism of KIRs in HIV subjects remains limited.

A significant body of evidence suggests that the innate immune system plays an important role in susceptibility to HIV infection and disease progression. For this reason, genetic polymorphisms in genes of innate immune pathways may also influence the course of the disease. In the present study, we focused our attention on several key genes involved in regulation of the innate immune response. We employed ultra-high multiplex PCR on an Ion Torrent next-generation sequencing platform to sequence 23 innate immune genes of 94 individuals with HIV/AIDS. These data were then used to identify potential associations of SNPs with clinical parameters and disease progression. Of the 649 SNPs identified in this study, SNPs which associated with viral load were identified in the following genes at the respective chromosomal positions:* IL15RA* (rs2229135; chr10:5995052),* TLR7* (rs179008, chrX:12903659),* TRIM5* (rs11601507; chr11:5701074),* KIR2DL1* (rs77397437; chr19:55286864), and* KIR2DL3* (chr19:55251049). Further analysis revealed that the C/T genotype of* IL15RA* (rs2229135; chr10:5995052), the A/T genotype of* TLR7* (rs179008; chrX:12903659), the A/C genotype of* KIR2DL1* (rs77397437; chr19:55286864), the A/G genotype of* KIR2DL3* (chr19:55251049), and the C/A genotype of* TRIM5* (rs11601507; chr11:5701074) were associated with increased viral load in HIV cases. Furthermore, two SNPs; one in* OAS2* (rs2072137; chr12:113440921) and the other in* OAS3* (chr12:113376388), were identified that associated with progression of HIV infection. Additionally, three SNPs associated with disease progression included* TRIM5* (rs11038628; chr11:5688940;),* KIR2DS4* (chr19:55358734) as well as the SNP in* IL15RA* (rs2229135; chr10:5995052). These data further support the role of the innate immune response in maintaining viral persistence and disease progression in HIV-infected individuals.

## 2. Materials and Methods

### 2.1. Subjects

Ninety-four cases (34 female and 60 male) hospitalized at the Republican Center for AIDS Prophylaxis and Prevention, Republic of Tatarstan, were enrolled in this study. Diagnosis of HIV infection was established based on presence of anti-HIV antibodies using ELISA and western blot methods. Cheek swabs were collected from all HIV-infected cases for genetic analysis. The Institutional Review Board of the Kazan Federal University approved this study and informed consent was obtained from each study subject according to the guidelines approved under this protocol (article 20, Federal Law “Protection of Health Right of Citizens of Russian Federation” N323-FZ, 11.21.2011). The clinical characteristics of HIV cases are summarized in Table 1.

### 2.2. Ampliseq Analysis

Genomic DNA was isolated using QIAamp DNA Mini Kit (Qiagen, Hilden, Germany) according to manufacturer's instructions. A DNA sample from each subject was used for sequencing by Ampliseq ultra-high multiplex PCR (IonAmpliseq Library kit; Life Technologies, Carlsbad, CA). Custom primer sets were designed using the Ion Ampliseq Designer tool (Life Technologies). The respective genes sequenced in this study are summarized in Table 2. The library after purification, equalization, and the template prep was processed for the Ion 318 Chip on the Ion OneTouch system (Thermo Fisher Scientific, Waltham, MA). Final sequencing was conducted using the Ion PGM Next-Generation Sequencing Platform (Thermo Fisher Scientific, Waltham, MA).

Reads from Ampliseq amplicon sequencing were aligned to the reference human genome (NCBI37/hg19) using the Bowtie2 alignment tool [19]. Single nucleotide variants were called at each amplicon using SAMtools and BCFtools [20]. Possible genotypes and their likelihoods were computed at each position with SAMtools mpileup, from which variants and genotypes were called using BCFtools [19].

## 3. Statistical Analysis

Six hundred forty-nine polymorphisms were identified across genotypes of 94 of the HIV samples using Ampliseq sequencing technology. Analyses of identified polymorphisms revealed 261 SNPs exhibited a minor allele frequency of 3.5% [94 HIV samples + CF1 + CFS2 + USCTR = 160]. Next, 261 SNPs were examined for association with clinical parameters including sCD14, viral load, and progressor status.

To identify association between genotype and sCD14 or viral load measures, a simple linear model approach was performed between each clinical parameter and the genotypes of the HIV samples. These *p* values were then adjusted using a multiple testing correction (false discovery rate) [21], and any adjusted *p* values with *p* < 0.05 were considered statistically significant.

Simple Chi-squared tests were performed to establish any relation between the genotypes of the 94 HIV samples and their progressor status. The *p* values of the 261 Chi-squared tests were not adjusted.

## 4. Results

### 4.1. Clinical Presentation of HIV Cases

Diagnosis of HIV infection was established by detection of anti-HIV antibodies using ELISA and western blot methods. The following criteria were used for patient enrollment: (a) detection of anti-HIV antibody; (b) CD4 counts ≥ 350 cell/*μ*L at the time of enrollment; and (c) absence of opportunistic infections at the time of enrollment. Subjects were followed up for five years after enrollment to determine progression of the disease.

A total of 94 subjects were enrolled in this study (Table 1): 34 female (36.2%) and 60 male (63.8%) HIV-infected cases with an average age 35.6 years. Mode of transmission included 39 sexual contact cases (41.5%) and 55 IV drug users cases (58.5%). Enrolled cases were at clinical stage 3 (30 cases; 31.9%), stage 4A (19 cases; 20.2%), and stage 4B (45 cases; 47.9%). Thirty-nine cases (41.5%) received antiviral treatment, while 55 cases (58.5%) remained without virus-specific therapy. Antiviral treatment included nucleoside analogs, nonnucleoside reverse transcriptase inhibitors, and protease inhibitors. Average HIV RNA viral load was 39925.9 copies/mL; 55 cases (58.5%) had a viral load higher than 250 copies/mL and the remaining 39 cases (41.5%) had an undetectable viral load or less than 250 copies/mL.

Based on CD4 counts and the rate of their decline, all cases were separated into two groups: group 1 “typical progressors” and group 2 “slow progressors.” Group 1, typical progressors, was characterized by a decline of CD4 counts >50 cells/*μ*L annually with overall CD4 counts < 350 cells/*μ*L during the five years of observation [22–24]. During this five-year period, subjects in group 1 lost on average 250 cells/*μ*L CD4 cells. Group 2, slow progressors, was characterized by <50 cells/*μ*L annual decline in CD4 counts with overall CD4 counts > 350 cells/*μ*L. Group 1 included 55 cases (58.5%), while group 2 contained 39 cases (41.5%).

### 4.2. Ampliseq Analysis

Twenty-three genes were selected for Ampliseq analysis (Table 2). The selected genes were chosen as mediators of innate immune responses and their corresponding intracellular pathways. Using the Ampliseq approach, complete sequences of each gene of interest were obtained allowing the identification of known, as well as previously unidentified SNPs. A total of 649 SNPs were identified, which were not present in the reference genome or in any of the 48 non-HIV controls. Correlation analyses were performed between each of the identified SNPs and antibody titer to LPS and LPS glycolipid, CD4 counts, viral load, and sCD14 level (Table 3). Data on serum level of sCD14 was available for 59 HIV cases.

Significant correlations between viral load and following SNPs were observed:* IL15RA* (rs2229135 chr10:5995052),* TLR7* (rs179008; chrX:12903659),* TRIM5* (rs11601507; chr11:5701074),* KIR2DL1* (rs77397437; chr19:55286864), and* KIR2DL3* (chr19:55251049). Specifically, our analyses revealed that the C/T genotype of* IL15RA* (rs2229135; chr10:5995052), the A/T genotype of* TLR7* (rs179008; chrX:12903659), the A/C genotype of* KIR2DL1* (rs77397437; chr19:55286864), the A/G genotype of* KIR2DL3* (chr19:55251049), and the C/A genotype of* TRIM5* (rs11601507; chr11:5701074) were associated with high viral loads.

Analyses were then performed to identify potential correlations between SNPs and disease progression (Table 4).* OAS2* (rs2072137; chr12:113440921) and* OAS3* (chr12:113376388) SNPs showed significant correlation with disease progression. Also,* OAS3* A/A genotype (chr12:113376388) was associated with slow disease progression, while genotypes G/G and G/A were linked to “typical” disease progression. Additionally, the* OAS2* T/C and C/C genotypes (rs2072137; chr12:113440921) were associated with “typical” disease progression, while the T/T genotype presented with higher frequency in the slow progressors. Lastly, our analysis revealed a higher frequency of* TRIM5* C/T genotype (rs11038628; chr11:5688940), the* KIR2DS4* G/A and A/A genotypes (chr19:55358734), and* IL15RA* (rs8177743; chr10:5995052;) C/T genotype in the “typical” progressors.

## 5. Discussion

Without antiretroviral therapy, the majority of those infected with HIV develop uncontrolled viremia and undergo progressive immune impairment ultimately leading to AIDS within three years of their diagnosis [24, 25]. However, a small number of HIV-infected individuals are characterized by a slow progression of HIV infection where CD4 counts are maintained above 350 cells/*μ*L without the development of AIDS for decades [26–28]. Several mechanisms have been suggested to explain this phenomenon including viral mutation, immune response, and host genetic factors. Since the virus relies heavily on the host's cellular machinery for replication, initial studies have focused on the host's genetic factors in an effort to explain these observations. For instance, screening of genetic variants in selected genes led to the discovery of a 32 bp deletion (*CCR5Δ32*) in the C-C chemokine receptor type 5 (*CCR5*) gene, which codes for a coreceptor used by the virus to enter the host cells [29, 30]. It has been demonstrated that the* CCR5Δ32* genotype delays HIV progression in heterozygous individuals and prevents infection in homozygous individuals [31, 32]. Discovery of the* CCR5Δ32* genotype provided compelling evidence to support the role of host genetic factors in the pathogenesis of HIV and AIDS. Since then, many studies have been conducted to identify additional genetic markers that influence disease progression.

Using a combination of analyses including genome-wide association studies (GWAS), many SNPs have been identified that associate with different forms of HIV progression. For example, it has been shown that long-term nonprogressors have a higher frequency of the HLA B^*∗*^5701, B5703, and HLA B^*∗*^2705 alleles [28, 33, 34]. Furthermore, Pereyra et al. proposed that the B14, B52, Bw4, and Cw1402 haplotypes were protective [33, 35]. It has been suggested that the HLA-B allele is more protective against HIV-1 when compared to the HLA-A allele. Studies of HLA and killer-cell immunoglobulin-like receptors revealed that individuals expressing KIR2DS2 have a more rapidly declining CD4 population and progression to AIDS than those who do not [36]. Conversely, the genotype KIR3DS1/HLA-B Bw4-80I, which stimulates NK cell activation, has been shown to promote virus containment and delay progression to AIDS [37, 38].

In this study, we have analyzed the complete sequence of 23 innate immune genes from 94 individuals with HIV/AIDS. The genes studied were divided into three groups: one group pertained to NK cell function and included genes of KIR family as well as* IL15RA*. The second group encompassed genes coding for the intrinsic antiviral factors OAS, RNASEL, TLRs, and TRIMs. Finally, the third group included the downstream type I interferon response transcription factors TRIF, MYD88, TRAF6, and IRF7. The Ampliseq method employed in this study utilizes a high throughput sequencing approach to identify known as well as novel SNPs. Correlation analyses were performed between the 649 identified SNPs and clinical laboratory findings such as CD4 counts, anti-LPS antibody, anti-LPS glycolipid antibody, and viral load. Selection of clinical parameters was based on their role in disease progression and outcome. Changes in CD4 counts determine the course of HIV infection and establish a starting point for HAART [39]. Presence of anti-LPS and anti-LPS glycolipid antibodies reflect the integrity of gut epithelium, which is affected as HIV infection progresses [40]. Viral load is a diagnostic marker for HIV infection as well as being used to monitor disease progression and efficacy of HAART [41, 42].

We observed the following SNPs, at the respective chromosomal positions, to be associated with viral load:* IL15RA* (rs2229135; chr10:5995052),* TLR7* (rs179008; chrX:12903659),* TRIM5* (rs11601507; chr11:5701074),* KIR2DL1* (rs77397437; chr19:55286864), and* KIR2DL3* (chr19:55251049). The SNP rs2229135 of* IL15RA* occurs in the three prime untranslated region (3′-UTR) of the gene and thus does not result in an amino acid change. However, SNPs that occur in this region can influence translation efficiency, polyadenylation, localization, and mRNA stability of their respective gene [43, 44]. Given the role of IL15 signaling in NK cell function, altered translation of* IL15R*A may broadly impact NK-cell-mediated antiviral immunity. In contrast to the* IL15R*A polymorphism, the SNPs observed in* TLR7* and* TRIM5* both result in missense substitutions. The SNP rs179008 in* TRL7* leads to a nonsynonymous Gln11Leu substitution within exon of the gene. Previous studies have reported this polymorphism is associated with chronic hepatitis C virus infection and systemic lupus erythematosus [45, 46]. Additionally, in support of a role for this polymorphism in HIV and in further support of our observations, Beima-Sofie et al. reported an association between a the* TLR7* 32T (rs179008) allele and time to mortality in female infants infected [47]. The SNP rs11601507 in* TRIM5* leads to a nonsynonymous Val112Phe missense substitution; however, previous studies addressing the potential role for this SNP and HIV found no association [48, 49]. However, other SNPs identified in the* TRIM5* report that some SNPs in this gene may be protective [50]. Notwithstanding, our data support the notion that cellular mechanisms involving* TRIM5* and* TLR7* are important in controlling HIV infection.

Two significant SNPs were identified in two KIR genes that also associated with viral load-*KIR2DL1* (rs77397437; chr19:55286864) and* KIR2DL3* (chr19:55251049). The rs77397437 SNP in* KIR2DL1* leads to the synonymous substitution Pro206, while the previously nonannotated SNP* KIR2DL3* (chr19:55251049) occurs in an intron. Although other studies have identified KIR associations with HIV infection, to the best of our knowledge, our data represents the first association with these specific polymorphisms and HIV viral load.

Also occurring within introns, two additional SNPs showed significance when analyzed in relation to disease progression-*OAS2* (rs2072137; chr12:113440921) and* OAS3* (chr12:113376388). We observed the* OAS2* T/C and C/C genotypes (rs2072137; chr12:113440921) to be associated with “typical” disease progression; however, the T/T genotype presented with higher frequency in the slow progressors. It is well documented that SNPs residing within introns, or those upstream or downstream of genes, also have the capacity to be causal [51–54]. In fact, in a recent study, Farh and colleagues utilized a fine-mapping algorithm to analyze GWAS data for 21 autoimmune diseases and reported that approximately 90% of all causal variants map to noncoding regions [55]. They further reported that only 10–20% of causal SNPs directly alter recognizable transcription factor binding motifs.

Our results also provide evidence of an association between various KIRs and HIV progression. A large body of evidence suggests a role for KIR3DS1 and its HLA ligand Bw4 in protection from HIV infection and delayed HIV progression [56, 57]. For instance, Alter et al. demonstrated that KIR3DS1+ NK cells are more potent in killing HIV-infected Bw4-80I+ CD4 T cells [18]. Additionally, an epidemiological study by Martín et al. revealed an association between slower HIV progression and the expression of KIR3DL1 [58]. It has been suggested that KIR3DL1 could play a role in “educating” NK cells, with higher expression of this KIR, promoting the generation of a larger pool of functionally competent NK cells, thus establishing a more vigorous response towards infection [59, 60]. Previous data pertaining to SNPs in KIRs has been limited to demonstrating that the variations in KIR2DS3 were associated with sustained viral response to pegylated-IFN and ribavirin treatment in HIV cases coinfected with HCV [61]. In the present study, we identified SNPs in* KIR2DL1*,* KIR2DL3*, and* KIR2DS4* that associated with maintenance of viral load and disease progression. However, further studies will be required to firmly establish the relationship these SNPs and the course of the disease.

The role of TRIM5 in controlling HIV replication is well established [62–64]. Previous studies have shown that the presence of SNPs in* TRIM5* exon2 and linker regions attenuates its ability to control viral replication [65, 66]. Our data provides further support for a TRIM5 involvement in controlling HIV replication and disease progression. We identified two SNPs, (rs11601507; chr11:5701074) and (rs11038628; chr11:5688940), which have a greater frequency in HIV cases with high viral load and “typical” progression of the disease. Furthers studies of HIV cases with these SNPs may provide additional support for their potential application as biological markers of disease progression.

Perhaps our most intriguing observation was the association between viral load, disease progression, and IL15RA. IL15RA binds specifically to IL-15, which is required for the differentiation of NK cells, CD8+ lymphocytes, and memory CD8+ T cells [67, 68]. Importantly, Naora and Gougeon demonstrated that when used in vitro, IL-15 stimulates the proliferation of CD56+, CD16+, CD4+, and CD8+ T cells from HIV-infected individuals [69]. They also showed that IL-15 was more potent than IL-2 as a survival factor for CD56+ cells, and this effect was associated with upregulation of Bcl-2 expression. Taken together, these findings indicated that IL-15 plays a pivotal role in survival and proliferation of NK cells in control of HIV maintenance and disease propagation. Our SNP data with regard to the* IL15RA* identifies a potential mechanism for the regulation of NK cell activity in HIV-infected individuals, in addition to presence of KIR SNPs. However, these previously unreported observations would require further in vitro and clinical evaluation to confirm their involvement in HIV disease progression.

Our data on the association of SNPs in genes coding for OAS2 and OAS3 with disease progression represents an additional and potentially novel finding. The OAS enzymes are interferon-inducible proteins required for the activation of RNase L [70]. While they become activated by the transactivation responsive region of HIV-1 mRNA, OAS activation is prevented by viral Tat binding to TAR [71–73]. Furthermore, RNase L inhibitor is activated in HIV-1-infected cells downregulating the OAS/RNase L pathway [74]. Therefore, it is not surprising that OAS enzymes are activated in HIV-infected cells as part of innate antiviral response and the efficacy of antiviral defense will, in part, depend on the ability of infected cells to prevent OAS inhibition. Our data on association between HIV disease progression and SNPs in* OAS* genes provides additional evidence of a role for these genes in HIV progression; however, additional studies are needed to firmly establish importance of these SNPs in HIV pathogenesis.

The prevalence of a given allele, whether it is protective or causative, may contribute to the prevalence of a disease. The Global Minor Allele Frequency (MAF) of rs2229135 for* IL15RA* is 0.09 [75]. In contrast, the same allele has a frequency of 0.11 in African populations, 0.05 in East Asian populations, and 0.08 in European populations [75]. The MAP of rs2072137 for* OAS2* has minor allele frequency in America, East Asia, and Europe of 0.48, 0.38, and 0.42, respectively, but only 0.06 in African populations [75]. Even more striking, the MAF of rs179008 for* TLR7* is 0.12 and a minor allele frequency of 0.23 in European populations but is only 0.05 in Southern Asian populations and is virtually zero in East Asian populations [75]. Larger studies that investigate clinical presentations, such as disease progression, in concert with genetic screening, may provide further support for the role of these genes in the pathogenesis of HIV/AIDS.

In summary, we have identified multiple SNPs that associate with viral load in the following genes:* IL15RA* (rs2229135; chr10:5995052),* TLR7* (rs179008; chrX:12903659),* TRIM5* (rs11601507; chr11:5701074),* KIR2DL1* (rs77397437; chr19:55286864), and* KIR2DL3* (chr19:55251049). Furthermore, two SNPs,* OAS2* (rs2072137; chr12:113440921) and* OAS3* (chr12:113376388), were identified to associate with the progression of HIV infection. Finally, SNPs in three genes demonstrated associations with disease progression including* TRIM5* (rs11038628; chr11:5688940),* KIR2DS4* (chr19:55358734), and* IL15RA* (rs2229135; chr10:5995052). If confirmed by future studies, these data may reveal important therapeutic targets for vaccine development and provide a greater understanding of disease pathogenesis.

## 6. Conclusions

In conclusion, we identified association between previously unreported SNPs in IL15RA, TLR7, TRIM5,* KIR2DL1*, and* KIR2DL3* and an increased viral load. Additionally, novel SNPs that associated with progression from HIV infection to AIDS were identified in* OAS2* and* OAS3* genes. In contrast, other SNPs identified in* OAS2* and* OAS3* genes, as well as in the* TRIM5* and* KIR2DS4* genes, were associated with a slower progression of disease. Further studies will determine the significance of identified SNPs in pathogenesis of HIV infection and AIDS.

## Figures and Tables

**Table 1:** HIV case characteristics.

Characteristics		
Age (years)	35.6	24–62
Male/female	36/60	
Mode of transmission		
Sexual contact	41	
IV drug user	55	
Clinical stage of the disease (CDC)		
A1	18	
A2	26	
A3	2	
B1	9	
B2	13	
B3	7	
C1	4	
C2	8	
C3	8	
Antiviral treatment		
Yes	39	
No	57	
HIV RNA viral load >250 copies/ml		
yes	57	
No	39	
HIV RNA load, average copies/ml	39925.9	
CD4 cell count/ml	413.9	
Long-term nonprogressors	53	
Rapid progressors	46	

**Table 2:** Genes selected.

	Gene	Function
1	OAS1	Induced by INF, essential innate immune response protein, member of the 2-5A synthase family
2	OAS2	Induced by INF, essential innate immune response protein, member of the 2-5A synthase family
3	OAS3	Induced by INF, essential innate immune response protein, member of the 2-5A synthase family
4	IL15RA	Binds specifically to IL15 (1993, *Mammalian Genome* **4** (8): 435-9)
5	TLR3	Pathogen pattern recognition receptor; binds to dsRNA
6	TLR4	Pathogen pattern recognition receptor; binds to LPS
7	TLR7	Pathogen pattern recognition receptor; binds to ssRNA
8	TLR8	Pathogen pattern recognition receptor; binds to ssRNA
9	TLR9	Pathogen pattern recognition receptor; binds to DNA
10	KIR2DL1	Inhibitory receptor, HLA-C alleles ligand
11	KIR2DL3	Inhibitory receptor, HLA-C alleles (HLA-Cw1, HLA-Cw3, and HLA-Cw7) ligands
12	KIR2DL4	Inhibitory receptor, expressed in endosome, HLA-G ligand (Front Immunol. 2012 Aug 20; 3:258. doi: 10.3389/fimmu.2012.00258. eCollection 2012)
13	KIR2DS4	Activation receptor; HLA-Cw4 ligand
14	KIR3DL1	Inhibitory receptor; HLA Bw4 ligand
15	KIR3DL2	Inhibitory receptor; HLA-A ligand
16	KIR3DL3	Inhibitory receptor, ligand unknown (Korean J Hematol. 2011 Dec; 46(4): 216-28. Doi)
17	IRF7	Activates transcription of INF; exclusively expressed in lymphoid tissue
18	TRIM5	Retrovirus restriction factor; binds to virus capsid and presents uncoating
19	TRIM21	Involved into intracellular antibody mediated proteolysis
20	RANSEL	Interferon-induced ribonuclease; degrade RNA cellular and viral
21	MYD88	Adapter protein involved in the Toll-like receptor and IL-1 receptor signaling pathway
22	TRIF	TLR adaptor protein
23	TRAF6	Member of the TNF receptor associated factor; TLR adaptor protein

**Table 3:** Correlation between viral load and SNPs.

Gene	Chromosome	Position	Reference genotype	SNP		*p* value
				Seq	Number	
IL15RA	10	5995052	C	CT	6	3.36*E* − 06
				CC	89	

KIR2DL1	19	55286864	A	AC	5	0.01
				AA	90	

KIR2DL3	19	55251049	A	AG	8	0.04
				AA	87	

TLR7	X	12903659	A	TT	5	0.02
				AT	10	
				AA	80	

TRIM5	11	5701074	C	CA	6	0.04
				CC	89	

**Table 4:** Correlation between disease progression and SNPs.

Gene	Chromosome	Position	Reference genotype	SNP		*p* value
				Seq	number	
TRIM5	11	5688940	C	CT	15	0.1005
				CC	80	

OAS2	12	113440921	T			0.01936

OAS3	12	113376388	G	AA	7	0.04115
				GA	6	
				GG	82	

KIR2DS4	19	55358734	G	GA	62	0.09112
				GG	33	

IL15RA	10	5995052	C	CT	6	0.07974
				CC	89	

## References

[1] Murray H. W., Godbold J. H., Jurica K. B., Roberts R. B. (1989). Progression to AIDS in patients with lymphadenopathy or AIDS-related complex: reappraisal of risk and predictive factors. *The American Journal of Medicine*.

[2] Okulicz J. F., Marconi V. C., Landrum M. L. (2009). Clinical outcomes of elite controllers, viremic controllers, and long-term nonprogressors in the US department of defense HIV natural history study. *Journal of Infectious Diseases*.

[3] Dolan M. J., Kulkarni H., Camargo J. F. (2007). CCL3L1 and CCR5 influence cell-mediated immunity and affect HIV-AIDS pathogenesis via viral entry-independent mechanisms. *Nature Immunology*.

[4] Ahuja S. K., Kulkarni H., Catano G. (2008). CCL3L1-CCR5 genotype influences durability of immune recovery during antiretroviral therapy of HIV-1-infected individuals. *Nature Medicine*.

[5] Tomescu C., Abdulhaqq S., Montaner L. J. (2011). Evidence for the innate immune response as a correlate of protection in human immunodeficiency virus (HIV)-1 highly exposed seronegative subjects (HESN). *Clinical and Experimental Immunology*.

[6] Cheney K. M., Mcknight Á. (2010). Interferon-alpha mediates restriction of human immunodeficiency virus type-1 replication in primary human macrophages at an early stage of replication. *PLoS ONE*.

[7] Lombardi V. C., Khaiboullina S. F. (2014). Plasmacytoid dendritic cells of the gut: relevance to immunity and pathology. *Clinical Immunology*.

[8] Papadopoulos A. I., Ferwerda B., Antoniadou A. (2010). Association of toll-like receptor 4 Asp299Gly and Thr399Ile polymorphisms with increased infection risk in patients with advanced HIV-1 infection. *Clinical Infectious Diseases*.

[9] Netea M. G., Van der Graaf C. A. A., Vonk A. G., Verschueren I., Van der Meet J. W. M., Kullberg B. J. (2002). The role of toll-like receptor (TLR) 2 and TLR4 in the host defense against disseminated candidiasis. *Journal of Infectious Diseases*.

[10] Ferwerda B., McCall M. B., Alonso S. (2007). TLR4 polymorphisms, infectious diseases, and evolutionary pressure during migration of modern humans. *Proceedings of the National Academy of Sciences of the United States of America*.

[11] Carvalho A., Pasqualotto A. C., Pitzurra L., Romani L., Denning D. W., Rodrigues F. (2008). Polymorphisms in toll-like receptor genes and susceptibility to pulmonary aspergillosis. *Journal of Infectious Diseases*.

[12] Soriano-Sarabia N., Vallejo A., Ramirez-Lorca R. (2008). Influence of the Toll-like receptor 9 1635A/G polymorphism on the CD4 count, HIV viral load, and clinical progression. *Journal of Acquired Immune Deficiency Syndromes*.

[13] Vallejo A., Valladares A., De Felipe B. (2005). High thymic volume is associated with viral replication and immunologic impairment only early after HAART interruption in chronic HIV infection. *Viral Immunology*.

[14] LaBonte M. L., McKay P. F., Letvin N. L. (2006). Evidence of NK cell dysfunction in SIV-infected rhesus monkeys: impairment of cytokine secretion and NKG2C/C2 expression. *European Journal of Immunology*.

[15] Alter G., Teigen N., Davis B. T. (2005). Sequential deregulation of NK cell subset distribution and function starting in acute HIV-1 infection. *Blood*.

[16] Mavilio D., Lombardo G., Kinter A. (2006). Characterization of the defective interaction between a subset of natural killer cells and dendritic cells in HIV-1 infection. *Journal of Experimental Medicine*.

[17] De Maria A., Fogli M., Costa P. (2003). The impaired NK cell cytolytic function in viremic HIV-1 infection is associated with a reduced surface expression of natural cytotoxicity receptors (NKp46, NKp30 and NKp44). *European Journal of Immunology*.

[18] Alter G., Martin M. P., Teigen N. (2007). Differential natural killer cell-mediated inhibition of HIV-1 replication based on distinct KIR/HLA subtypes. *Journal of Experimental Medicine*.

[19] Langmead B., Salzberg S. L. (2012). Fast gapped-read alignment with Bowtie 2. *Nature Methods*.

[20] Li H., Handsaker B., Wysoker A. (2009). The sequence alignment/map format and SAMtools. *Bioinformatics*.

[21] Benjamini Y., Hochberg Y. (1995). Controlling the false discovery rate: a practical and powerful approach to multiple testing. *Journal of the Royal Statistical Society. Series B. Methodological*.

[22] Margolick J. B., Muñoz A., Vlahov D. (1992). Changes in T-lymphocyte subsets in intravenous drug users with HIV-1 infection. *The Journal of the American Medical Association*.

[23] Hughes M. D., Stein D. S., Gundacker H. M., Valentine F. T., Phair J. P., Volberding P. A. (1994). Within-subject variation in CD4 lymphocyte count in asymptomatic human immunodeficiency virus infection: implications for patient monitoring. *Journal of Infectious Diseases*.

[24] Audigé A., Taffé P., Rickenbach M. (2010). Low postseroconversion CD4 count and rapid decrease of CD4 density identify HIV+ fast progressors. *AIDS Research and Human Retroviruses*.

[25] Farzadegan H., Henrard D. R., Kleeberger C. A. (1996). Virologic and serologic markers of rapid progression to AIDS after HIV-1 seroconversion. *Journal of Acquired Immune Deficiency Syndromes and Human Retrovirology*.

[26] Sheppard H. W., Lang W., Ascher M. S., Vittinghoff E., Winkelstein W. (1993). The characterization of non-progressors: long-term HIV-1 infection with stable CD4+ T-cell levels. *AIDS*.

[27] Bakari M., Urassa W., Mhalu F., Biberfeld G., Pallangyo K., Sandström E. (2008). Slow progression of HIV-1 infection in a cohort of antiretroviral naïve hotel workers in Dar es Salaam, Tanzania as defined by their CD4 cell slopes. *Scandinavian Journal of Infectious Diseases*.

[28] Fellay J., Shianna K. V., Ge D. (2007). A whole-genome association study of major determinants for host control of HIV-1. *Science*.

[29] Liu R., Paxton W. A., Choe S. (1996). Homozygous defect in HIV-1 coreceptor accounts for resistance of some multiply-exposed individuals to HIV-1 infection. *Cell*.

[30] Carrington M., Dean M., Martin M. P., O'Brien S. J. (1999). Genetics of HIV-1 infection: chemokine receptor CCR5 polymorphism and its consequences. *Human Molecular Genetics*.

[31] Quillent C., Oberlin E., Braun J. (1998). HIV-1-resistance phenotype conferred by combination of two separate inherited mutations of CCR5 gene. *Lancet*.

[32] Stewart G. J., Ashton L. J., Biti R. A. (1997). Increased frequency of CCR-5 delta 32 heterozygotes among long-term non-progressors with HIV-1 infection. The Australian Long-Term Non-Progressor Study Group. *AIDS*.

[33] Pereyra F., Jia X., McLaren P. J., Telenti A., De Bakker P. I. W., Walker B. D. (2010). The major genetic determinants of HIV-1 control affect HLA class I peptide presentation. *Science*.

[34] Migueles S. A., Sabbaghian M. S., Shupert W. L. (2000). HLA B∗5701 is highly associated with restriction of virus replication in a subgroup of HIV-infected long term nonprogressors. *Proceedings of the National Academy of Sciences of the United States of America*.

[35] Stern M., Czaja K., Rauch A. (2012). HLA-Bw4 identifies a population of HIV-infected patients with an increased capacity to control viral replication after structured treatment interruption. *HIV Medicine*.

[36] Gaudieri S., DeSantis D., McKinnon E. (2005). Killer immunoglobulin-like receptors and HLA act both independently and synergistically to modify HIV disease progression. *Genes & Immunity*.

[37] Qi Y., Martin M. P., Gao X. (2006). KIR/HLA pleiotropism: protection against both HIV and opportunistic infections. *PLoS Pathogens*.

[38] Jiang Y., Chen O., Cui C. (2013). KIR3DS1/L1 and HLA-Bw4-80I are associated with HIV disease progression among HIV typical progressors and long-term nonprogressors. *BMC Infectious Diseases*.

[39] Lepri A. C., Phillips A. N., Monforte A. D. (2001). When to start highly active antiretroviral therapy in chronically HIV-infected patients: evidence from the ICONA study. *AIDS*.

[40] Kelly P., Shawa T., Mwanamakondo S. (2010). Gastric and intestinal barrier impairment in tropical enteropathy and HIV: limited impact of micronutrient supplementation during a randomised controlled trial. *BMC Gastroenterology*.

[41] Duong M., Piroth L., Peytavin G. (2001). Value of patient self-report and plasma human immunodeficiency virus protease inhibitor level as markers of adherence to antiretroviral therapy: relationship to virologic response. *Clinical Infectious Diseases*.

[42] Schmit J.-C., Weber B. (1998). Recent advances in antiretroviral therapy and HIV infection monitoring. *Intervirology*.

[43] Barrett L. W., Fletcher S., Wilton S. D. (2012). Regulation of eukaryotic gene expression by the untranslated gene regions and other non-coding elements. *Cellular and Molecular Life Sciences*.

[44] Pichon X., Wilson L. A., Stoneley M. (2012). RNA binding protein/RNA element interactions and the control of translation. *Current Protein and Peptide Science*.

[45] Askar E., Ramadori G., Mihm S. (2010). Toll-like receptor 7 rs179008/Gln11Leu gene variants in chronic hepatitis C virus infection. *Journal of Medical Virology*.

[46] dos Santos B. P., Valverde J. V., Rohr P. (2012). TLR7/8/9 polymorphisms and their associations in systemic lupus erythematosus patients from southern Brazil. *Lupus*.

[47] Beima-Sofie K. M., Bigham A. W., Lingappa J. R. (2013). Toll-like receptor variants are associated with infant HIV-1 acquisition and peak plasma HIV-1 RNA level. *AIDS*.

[48] Goldschmidt V., Bleiber G., May M., Martinez R., Ortiz M., Telenti A. (2006). Role of common human TRIM5*α* variants in HIV-1 disease progression. *Retrovirology*.

[49] Javanbakht H., An P., Gold B. (2006). Effects of human TRIM5*α* polymorphisms on antiretroviral function and susceptibility to human immunodeficiency virus infection. *Virology*.

[50] Price H., Lacap P., Tuff J. (2010). A TRIM5*α* exon 2 polymorphism is associated with protection from HIV-1 infection in the Pumwani sex worker cohort. *AIDS*.

[51] Kimchi-Sarfaty C., Oh J. M., Kim I.-W. (2007). A ‘silent’ polymorphism in the MDR1 gene changes substrate specificity. *Science*.

[52] Li G., Pan T., Guo D., Li L. (2014). Regulatory variants and disease: the E-Cadherin −160C/A SNP as an example. *Molecular Biology International*.

[53] Al-Haggar M., Madej-Pilarczyk A., Kozlowski L. (2012). A novel homozygous p.Arg527Leu LMNA mutation in two unrelated Egyptian families causes overlapping mandibuloacral dysplasia and progeria syndrome. *European Journal of Human Genetics*.

[54] Cordovado S. K., Hendrix M., Greene C. N. (2012). CFTR mutation analysis and haplotype associations in CF patients. *Molecular Genetics and Metabolism*.

[55] Farh K. K.-H., Marson A., Zhu J. (2015). Genetic and epigenetic fine mapping of causal autoimmune disease variants. *Nature*.

[56] Barber L. D., Percival L., Arnett K. L., Gumperz J. E., Chen L., Parham P. (1997). Polymorphism in the *α*1 Helix of the HLA-B Heavy Chain Can Have an Overriding Influence on Peptide-Binding Specificity. *Journal of Immunology*.

[57] Ravet S., Scott-Algara D., Bonnet E. (2007). Distinctive NK-cell receptor repertoires sustain high-level constitutive NK-cell activation in HIV-exposed uninfected individuals. *Blood*.

[58] Martín R., Carvalho J., Ibeas E., Hernández M., Ruiz-Gutierrez V., Nieto M. L. (2007). Acidic triterpenes compromise growth and survival of astrocytoma cell lines by regulating reactive oxygen species accumulation. *Cancer Research*.

[59] Carrington M., Alter G. (2012). Innate immune control of HIV. *Cold Spring Harbor Perspectives in Medicine*.

[60] Eller M. A., Koehler R. N., Kijak G. H. (2011). Human Immunodeficiency virus type 1 infection is associated with increased NK cell polyfunctionality and higher levels of KIR3DL1 + NK cells in ugandans carrying the HLA-B Bw4 motif. *Journal of Virology*.

[61] Keane C., O'Shea D., Reiberger T. (2013). Variation in both IL28B and KIR2DS3 genes influence pegylated interferon and ribavirin hepatitis C treatment outcome in HIV-1 co-infection. *PLoS ONE*.

[62] Wu W., Zheng N., Wang Y., Fung J. J., Lu L., Qian S. (2006). Immune regulatory activity of liver-derived dendritic cells generated in vivo. *Microsurgery*.

[63] Sebastian S., Luban J. (2005). TRIM5*α* selectively binds a restriction-sensitive retroviral capsid. *Retrovirology*.

[64] Stremlau M., Perron M., Lee M. (2006). Specific recognition and accelerated uncoating of retroviral capsids by the TRIM5*α* restriction factor. *Proceedings of the National Academy of Sciences of the United States of America*.

[65] Nakajima T., Nakayama E. E., Kaur G. (2009). Impact of novel TRIM5[alpha] variants, Gly110Arg and G176del, on the anti-HIV-1 activity and the susceptibility to HIV-1 infection. *AIDS*.

[66] Nakayama E. E., Nakajima T., Kaur G. (2013). A naturally occurring single amino acid substitution in human TRIM5*α* linker region affects its anti-HIV type 1 activity and susceptibility to HIV type 1 infection. *AIDS Research and Human Retroviruses*.

[67] Mingari M. C., Ponte M., Cantoni C. (1997). HLA-class I-specific inhibitory receptors in human cytolytic T lymphocytes: molecular characterization, distribution in lymphoid tissues and co-expression by individual T cells. *International Immunology*.

[68] Li S., Qi X., Gao Y. (2010). IL-15 increases the frequency of effector memory CD8^+^ T cells in rhesus monkeys immunized with HIV vaccine. *Cellular and Molecular Immunology*.

[69] Naora H., Gougeon M.-L. (1999). Enhanced survival and potent expansion of the natural killer cell population of HIV-infected individuals by exogenous interleukin-15. *Immunology Letters*.

[70] Silverman R. H. (2007). Viral encounters with 2′,5′-oligoadenylate synthetase and RNase L during the interferon antiviral response. *Journal of Virology*.

[71] Maitra R. K., McMillan N. A. J., Desai S. (1994). HIV-1 TAR RNA has an intrinsic ability to activate interferon-inducible enzymes. *Virology*.

[72] Sengupta D. N., Silverman R. H. (1989). Activation of interferon-regulated, dsRNA-dependent enzymes by human immunodeficiency virus-1 leader RNA. *Nucleic Acids Research*.

[73] Schroder H. C., Ugarkovic D., Wenger R., Reuter P., Okamoto T., Muller W. E. G. (1990). Binding of Tat protein to TAR region of human immunodeficiency virus type 1 blocks TAR-mediated activation of (2′-5′)oligoadenylate synthetase. *AIDS Research and Human Retroviruses*.

[74] Martinand C., Montavon C., Salehzada T., Silhol M., Lebleu B., Bisbal C. (1999). RNase L inhibitor is induced during human immunodeficiency virus type 1 infection and down regulates the 2-5A/RNase L pathway in human T cells. *Journal of Virology*.

[75] The 1000 Genomes Project Consortium (2015). A global reference for human genetic variation. *Nature*.

